# A study on the correlations between acoustic speech variables and bradykinesia in advanced Parkinson's disease

**DOI:** 10.3389/fneur.2023.1213772

**Published:** 2023-07-18

**Authors:** Francesco Cavallieri, Giulia Di Rauso, Annalisa Gessani, Carla Budriesi, Valentina Fioravanti, Sara Contardi, Elisa Menozzi, Serge Pinto, Elena Moro, Francesca Antonelli, Franco Valzania

**Affiliations:** ^1^Neurology Unit, Neuromotor and Rehabilitation Department, Azienda USL-IRCCS di Reggio Emilia, Reggio Emilia, Italy; ^2^Department of Biomedical, Metabolic and Neural Sciences, University of Modena and Reggio Emilia, Modena, Italy; ^3^Neurology, Neuroscience Head Neck Department, Azienda Ospedaliero-Universitaria di Modena, Modena, Italy; ^4^IRCCS Istituto delle Scienze Neurologiche di Bologna, Neurologia e Rete Stroke Metropolitana, Ospedale Maggiore, Bologna, Italy; ^5^Department of Clinical and Movement Neurosciences, UCL Queen Square Institute of Neurology, London, United Kingdom; ^6^Aix Marseille Univ, CNRS, LPL, Aix-en-Provence, France; ^7^Grenoble Alpes University, Division of Neurology, Centre Hospitalier Universitaire de Grenoble, Grenoble Institute of Neuroscience, Grenoble, France

**Keywords:** acoustic, bradykinesia, dysarthria, Parkinson's disease, speech

## Abstract

**Background:**

Very few studies have assessed the presence of a possible correlation between speech variables and limb bradykinesia in patients with Parkinson's disease (PD). The objective of this study was to find correlations between different speech variables and upper extremity bradykinesia under different medication conditions in advanced PD patients.

**Methods:**

Retrospective data were collected from a cohort of advanced PD patients before and after an acute levodopa challenge. Each patient was assessed with a perceptual-acoustic analysis of speech, which included several quantitative parameters [i.e., maximum phonation time (MPT) and intensity (dB)]; the Unified Parkinson's Disease Rating Scale (UPDRS) (total scores, subscores, and items); and a timed test (a tapping test for 20 s) to quantify upper extremity bradykinesia. Pearson's correlation coefficient was applied to find correlations between the different speech variables and the tapping rate.

**Results:**

A total of 53 PD patients [men: 34; disease duration: 10.66 (SD 4.37) years; age at PD onset: 49.81 years (SD 6.12)] were included. Levodopa intake increased the MPT of sustained phonation (*p* < 0.01), but it reduced the speech rate (*p* = 0.05). In the defined-OFF condition, MPT of sustained phonation positively correlated with both bilateral mean (*p* = 0.044, *r*-value:0.299) and left (*p* = 0.033, *r*-value:0.314) tapping. In the defined-ON condition, the MPT correlated positively with bilateral mean tapping (*p* = 0.003), left tapping (*p* = 0.003), and right tapping (*p* = 0.008).

**Conclusion:**

This study confirms the presence of correlations between speech acoustic variables and upper extremity bradykinesia in advanced PD patients. These findings suggest common pathophysiological mechanisms.

## Introduction

Speech alterations are very common in Parkinson's disease (PD) and are reported in 70–90% of patients (1, 2). Hypokinetic dysarthria is the most frequent manifestation and can emerge at any stage of the disease, but it particularly aggravates in the later stages, causing a progressive loss of communication and leading to social isolation (3). Based on the perceptual analysis, hypokinetic dysarthria is characterized by harsh, breathy voice quality, reduced variability of pitch and loudness, reduced stress, imprecise consonant articulation, and short rushes of speech interrupted by inappropriate periods of silence (4).

In recent years, the acoustic analysis of speech has become an important tool in the study of PD and other movement disorders, allowing for the quantification of the alterations in the different dimensions of speech production (5–9).

Mixed results have been reported regarding the effects of dopaminergic treatment on speech acoustic variables in PD patients (6, 10, 11). This inconsistency in results could be secondary to the complex pathophysiology of speech alterations in PD that involve both dopaminergic and non-dopaminergic (i.e., cholinergic) pathways (10).

Besides speech alterations, PD is mainly characterized by well-known cardinal motor features, including bradykinesia, rigidity, tremor, and postural instability (12). According to the MDS Clinical Diagnostic Criteria for PD, bradykinesia is defined as “slowness of movement and decrement in amplitude or speed (or progressive hesitations/halts) as movements are continued (13).” Bradykinesia may impair the fine motor movements, which are usually demonstrated in PD patients during rapid alternating movements of the fingers, hands, or feet as a progressive reduction of speed and motion amplitude (14). Upper extremity bradykinesia can be clinically evaluated by using finger tapping, hand movements, and pronation-supination movements (13). It has been proposed that bradykinesia may result from a failure of basal ganglia output to reinforce the cortical mechanisms that prepare and execute the commands to move (15). This leads to particular difficulties with self-paced movements, prolonged reaction times, and abnormal pre-movement EEG activity (15). In PD patients, movement amplitude is disproportionally more affected than movement speed in the OFF-medication condition. Levodopa normalizes movement speed to a greater extent than movement amplitude, suggesting that movement speed and amplitude may be associated with partially separate mechanisms (16, 17). To date, prevailing evidence have indicated that hypokinetic dysarthria is related to axial motor symptoms, while only a few studies have documented an association between a speech disorder and limb bradykinesia in PD (4, 18–22). In this setting, very few studies have quantitatively assessed the possible correlations between speech acoustic variables and upper limb bradykinesia, including the presence of possible similarities in terms of response to levodopa (18, 19, 23). In addition, as previously reported, some features of hypokinetic dysarthria may respond to dopaminergic treatment (6) raising the doubt that hypokinetic dysarthria in PD should not be considered tout court an axial symptom but that it should be deconstructed by looking for different aspects either linked or not linked to axial and appendicular PD symptoms.

Based on these premises, the objective of this study was to verify if there are correlations between different speech variables and upper extremity bradykinesia in different medication conditions in advanced PD patients.

## Methods

### Participants

This study included retrospective data from a cohort of consecutive advanced PD patients admitted to the Neurology Unit of the OCB Hospital, Italy, for a preoperative evaluation before subthalamic nucleus deep brain stimulation (STN-DBS) surgery from 2012 to 2017.

The criteria of inclusion were PD diagnosis according to the MDS criteria (13), the presence of disabling motor complications (i.e., motor fluctuations or L-dopa-induced dyskinesia) not optimized with anti-PD medication; and age younger than 75 years (24, 25).

Patients with severe cognitive impairment or non-native Italian speakers were excluded from the analysis. This study was approved by the local ethics committee (Protocol number: 0031287/18), and written informed consent was obtained from participants according to the Declaration of Helsinki (26).

### Clinical assessment

Clinical evaluations were performed following the Core Assessment Program for Surgical Interventional Therapies in Parkinson's Disease (CAPSIT-PD) protocol (17).

Each patient was evaluated in the defined-OFF medication condition (after 12-h withdrawal of antiparkinsonian medication) and in the defined-ON medication condition (60 min after the administration of a 30% higher dose of the usual levodopa morning intake). Disease severity was assessed through the four parts of the Unified Parkinson's Disease Rating Scale (UPDRS) (27) and the Hoehn and Yahr (H&Y) scale staging system.

Furthermore, upper extremity bradykinesia was quantitatively assessed through a tapping timed test in accordance with the CAPSIT-PD protocol (17, 28) in the defined-OFF and -ON medication conditions. Each patient tapped alternatingly on two buttons (at a 20 cm distance) with the index finger by using the whole upper extremity for a defined fixed time (20 s). Each hand was tested twice, and the mean value of the tapping rate was reported. All tests were videotaped, and through the retrospective analysis of each video, it was possible to calculate with certainty the correct number of taps for each task. A free editing software (Wondershare Filmora 9) was used to analyze the video in slow motion. The retrospective analysis of the video was performed by a GDR blinded to both defined-ON and -OFF conditions.

The total amount of the dopaminergic treatment was determined using the L-dopa equivalent daily dose (LEDD) (29).

### Speech evaluation

Patients' speech was evaluated in both the defined-OFF and defined-ON medication conditions by two speech and language therapists (CB and AG) with expertise in phonetics and movement disorders related to speech disturbances. Each session of speech evaluation took place immediately at the end of each neurological examination. Evaluations were made in a quiet room. The speech was recorded using a digital voice recorder maintained at 20 cm from the patients' lips. The acoustic speech analysis was performed using the Praat software (30). The perceptual-acoustic analysis was retrospectively performed, with the speech and language therapists blinded to the patient's condition. The speech assessment protocol (6, 7) consisted of various tasks, including sustained production of the phoneme /a/ for as long as possible and performed three times, counting from 1 to 20, and an oral diadochokinesis task in which the participants produced the syllables /pa/, /ta/, /ka/ and the pseudoword /pataka/ as fast as they could with habitual pitch and loudness. The variables considered were maximum phonation time (MPT) [s], intensity [dB], a fraction of locally unvoiced frames, and a number of voice breaks (all these parameters were evaluated during sustained phonation tasks); speech rate [syllables/second] calculated during counting tasks; and irregular rhythm [presence of absence] and uncontrolled acceleration [presence of absence] evaluated during oral diadochokinesis tasks. Single-word intelligibility (calculated as the percentage of words correctly transcribed by the examiner among a set of 25 recorded words) was also calculated.

### Statistical analysis

Descriptive statistics were performed for clinical and acoustic variables; continuous variables were expressed as mean [standard deviation (SD)] and median (range), while frequencies and percentages were calculated for categorical variables. The variables were tested for normal distribution using the Kolmogorov-Smirnov test of normality. A *p*-value of < 0.05 was considered significant.

The primary outcome of the study was the possible correlation between the different speech variables and upper extremity bradykinesia quantified through the tapping test in different medication conditions. Concerning the tapping test, the following variables were selected for both defined-OFF and defined-ON medication conditions: mean value of the left hand; mean value of the right hand; and mean value of both hands ([mean value of the left hand + mean value of the right hand]/2).

The secondary outcome was the possible correlation between the levodopa-induced variation of speech variables and upper extremity bradykinesia, both calculated as follows: [(defined-ON value minus defined-OFF value)/defined-ON value] × 100. Positive scores denote an increase in speech variables or tapping rate. The correlation between speech variables and UPDRS motor scores and subscores was not included in the analysis because it was already performed in a previous study by our group (6). Considering that most variables were normally distributed, the Pearson correlation coefficient was applied. The correlation analyses, including speech and motor variables, were performed for one of the single conditions tested (defined-OFF, defined-ON). Statistical analyses were performed using the IBM SPSS Statistics software for Windows version 20.0 (IBM, Armonk, NY, USA).

## Results

### Demographic and clinical results

From a total of 66 consecutive advanced PD patients, 13 patients were excluded from the analyses for the following reasons: non-native Italian speakers (five patients), missing data (four patients), and severe cognitive impairment (four patients). The clinical and demographic characteristics of the remaining 53 patients are reported in Table 1.

**Table 1:** Demographic and clinical characteristics of PD patients.

**Variables**	**Total *n* = 53 *n* (%); mean (SD); median [range]**
**Age (years)**	60.47 (6.77); 62.00 [45.00–75.00]
**Sex**	
Men	34 (64.20 %)
Women	19 (35.80 %)
**Handedness**	
Left	5 (9.43 %)
Right	48 (90.57 %)
Age at PD onset (years)	49.81 (6.12); 50.00 [37.00–61.00]
Disease duration (years)	10.66 (4.37); 9.00 [5.00–25.00]
Duration of levodopa treatment (years)	7.50 (3.53); 7.00 [3.00–18.00]
UPDRS part I	2.58 (2.04); 2.00 [0.00–8.00]
UPDRS part IV	6.72 (2.47); 7.00 [2.00–12.00]
UPDRS dyskinesia subscore	2.48 (1.75); 2.00 [0.00–6.00]
LEDD (milligrams)	1087.32 (430.46); 1022.00 [225.00–2322.00]
Total CDRS score (0–28)	6.32 (4.60); 6.00 [0.00–21.00]
Axial CDRS subscore (0–12)	2.37 (2.18); 2.00 [0.00–9.00]

CDRS, Clinical Dyskinesia rating scale; LEDD, Levodopa Equivalent Daily Dose; UPDRS, Unified Parkinson's Disease Rating Scale; PD, Parkinson's Disease.

In the defined-ON medication condition, all motor scores, subscores, and tapping rates significantly improved, whereas only speech rate (*p* = 0.005) and MPT (*p* = 0.001) were influenced (Table 2).

**Table 2:** Changes of clinical variables and speech variables after levodopa intake.

**Variables**	***n*** **(%); mean (SD); median [range]**		**Percentage variation (%)**	***p*-Value**
	**Defined-OFF medication**	**Defined-ON medication**		
**Clinical Variables**				
UPDRS part II	21.52 (7.43); 21.00 [9.00–46.00]	8.48 (4.96); 8.50 [0.00–23.00]	−247.74 (289.31); −164.58 [−1500.00–8.70]	< 0.001
UPDRS part III	40.00 (12.23); 38.00 [15.00–87.00]	15.60 (7.44); 15.00 [1.00–38.00]	−237.39 (290.07); −145.45 [−1800.00–43.48]	< 0.001
H&Y	3.22 (0.80); 3.00 [2.00–5.00]	2.13 (0.67); 2.00 [0.00–4.00]	−58.58 (54.09); −50.00 [−300.00–0.00]	< 0.001
Speech subscore	3.31 (1.30); 3.00 [0.00–7.00]	1.69 (1.32); 2.00 [0.00–6.00]	−109.10 (93.31); −100.00 [−300.00–40.00]	< 0.001
PIGD subscore	9.42 (4.14); 9.00 [2.00–20.00]	3.71 (2.86); 3.00 [0.00–14.00]	−251.00 (275.95); −200.00 [−1500.00–14.29]	< 0.001
Speech item	1.67 (0.67); 2.00 [0.00–3.00]	0.92 (0.71); 1.00 [0.00–3.00]	−67.10 (61.81); −100.00 [−200.00–0.00]	< 0.001
Tapping left	48.07 (13.73); 47.50 [21.00–91.00]	62.87 (14.87); 63.50 [34.00–98.00]	21.77 (18.66); 21.69 [−42.18–63.33]	< 0.001
Tapping right	50.43 (14.53); 47.00 [27.00–95.50]	67.67 (15.74); 68.50 [36.00–101.00]	24.05 (18.14); 22.69 [−49.21–54.79]	< 0.001
Tapping bilateral	49.25 (13.84); 48.00 [25.00–93.25]	65.27 (14.78); 64.75 [36.00–93.25]	23.14 (17.59); 24.06 [−45.70–56.04]	< 0.001
**Speech variables**				
Speech rate (syll/sec)	4.90 (1.63); 4.75 [0.16–9.50]	4.47 (1.54); 4.63 [0.16–6.75]	−15.59 (29.77); −14.18 [−106.09–24.96]	0.005
Speech intelligibility (%)	86.67 (15.99); 92.00 [32.00–100.00]	87.49 (12.03); 90.00 [44.00–100.00]	1.75 (13.70);0.00 [−24.14–50.00]	0.360
Mean intensity of spontaneous speech (dB)	64.50 (6.73); 65.00 [46.00–76.00]	64.16 (7.89); 66.00 [49.00–79.00]	−1.12 (9.06);0.00 [−32.65–24.59]	0.635
F0 SD of spontaneous speech (Hz)	32.94 (19.07); 26.09 [9.22–91.42]	34.66 (20.34); 28.27 [9.87–126.55]	−7.39 (59.49); 5.02 [−282.76–72.55]	0.493
Maximum phonation time (MPT) (sec)	12.02 (5.12); 12.00 [2.00–26.00]	14.15 (5.49); 13.00 [4.00–26.00]	9.39 (35.46); 16.66 [−100.00–71.43]	0.001
Mean intensity of sustained phonation (dB)	72.22 (7.41); 74.00 [51.00–83.00]	70.90 (6.99); 72.00 [56.00–83.00]	−1.92 (8.46); −0.66 [−25.00–14.71]	0.137
Fraction of locally unvoiced frames (%)	3.06 (7.33); 0.47 [0.00–39.58]	2.40 (4.84); 0.15 [0.00–21.59]	−207.53 (571.74); 17.54 [−2522.27.−100.00]	0.500
Number of voice breaks	1.52 (3.09); 0.33 [0.00–15.59]	0.92 (1.74); 0.00 [0.00–8.66]	−200.90 (699.94); 13.84 [−2900.00–100.00]	0.205
Irregular rhythm				
Absence	42 (79.20%)	44 (83.00%)	N/A	0.246
Presence	11 (20.80%)	9 (17.00%)		
Uncontrolled acceleration				
Absence	41 (77.40%)	43 (81.10%)	N/A	0.229
Presence	12 (22.60%)	10 (18.90%)		

Tapping rate: Calculated as the number of taps in 20 s.

H&Y, Hoehn and Yahr scale; NA, not applicable; PIGD, postural instability and gait disorders; UPDRS, Unified Parkinson's Disease Rating Scale.

### Correlation between speech variables and upper extremity bradykinesia

The correlations between speech variables and upper extremity bradykinesia are reported in Table 3.

**Table 3:** Correlation between speech variables and upper extremity bradykinesia.

**Variables**		**Defined-OFF medication**			**Defined-ON medication**		
		**Left tapping**	**Right tapping**	**Bilateral tapping**	**Left tapping**	**Right tapping**	**Bilateral tapping**
Mean intensity of spontaneous speech (dB)	*r*	0.136	0.232	0.189	0.154	0.234	0.202
	*P*-value	0.368	0.121	0.208	0.308	0.118	0.179
F0 SD of spontaneous speech (Hz)	*r*	−0.099	−0.068	−0.084	0.059	0.092	0.079
	*P*-value	0.514	0.655	0.577	0.699	0.542	0.604
Maximum phonation time (MPT) (sec)	*r*	0.314	0.272	0.299	0.438	0.391	0.429
	*P*-value	**0.033**	0.067	**0.044**	**0.003**	**0.008**	**0.003**
Mean intensity of sustained phonation (dB)	*r*	0.149	0.235	0.197	0.099	0.128	0.118
	*P*-value	0.334	0.125	0.200	0.527	0.415	0.452
Fraction of locally unvoiced frames (%)	*r*	−0.191	−0.227	−0.213	−0.061	−0.052	−0.058
	*P*-value	0.238	0.159	0.186	0.707	0.749	0.720
Number of voice breaks	*r*	0.053	0.024	0.038	−0.149	0.006	−0.071
	*P*-value	0.746	0.885	0.814	0.358	0.971	0.665
Speech rate (syll/sec)	*r*	0.222	0.307	0.271	0.052	0.007	0.030
	*P*-value	0.139	**0.038**	0.069	0.735	0.962	0.847
Speech intelligibility (%)	*r*	−0.086	−0.122	−0.108	0.149	0.109	0.133
	*P*-value	0.633	0.497	0.550	0.323	0.470	0.378
Irregular rhythm	*r*	−0.230	−0.228	−0.232	0.062	0.056	0.068
	*P*-value	0.123	0.125	0.120	0.683	0.713	0.653
Uncontrolled acceleration	*r*	−0.090	−0.075	−0.088	−0.221	0.054	−0.085
	*P*-value	0.551	0.621	0.559	0.140	0.723	0.576

Bold values denote statistically significant.

In the defined-OFF medication condition, the MPT of sustained phonation correlated positively with bilateral mean tapping (*p* = 0.044, r-value:0.299). Analyzing the test for the single limb, the correlation remained significant for the left (*p* = 0.033, r-value:0.314), which presented a worse performance, while only a trend was detected on the right (*p* = 0.067, r-value:0.272).

In the defined-ON medication condition, the MPT correlated positively with bilateral mean tapping (*p* = 0.003, r-value:0.429); in this case, a significant correlation was maintained for both left (*p* = 0.003, r-value:0.438) and right tapping (*p* = 0.008, r-value:0.391).

In both the defined-OFF and -ON medication conditions, speech rate did not show a correlation with bradykinesia, with the exception of a weak significance (*p* = 0.038, r-value:0.307) for the right tapping in the defined-OFF condition. Nevertheless, these data were insufficient to support an unequivocal relationship between the two findings.

### Correlation between levodopa-induced changes in speech variables and upper extremity bradykinesia

Although both tapping tests, speech rate, and MPT significantly changed after levodopa intake, no significant correlations were found, which means that the effects of levodopa on these variables were not uniform (Table 4).

**Table 4:** Correlation between levodopa-induced changes of speech variables and upper extremity bradykinesia.

**Variables (% variation)**		**Right tapping**	**Left tapping**	**Bilateral tapping**
Mean intensity of spontaneous speech (dB)	*r*	0.198	0.092	0.151
	*P*-value	0.192	0.549	0.324
F0 SD of spontaneous speech (Hz)	*r*	0.014	0.038	0.025
	*P*-value	0.926	0.803	0.871
Maximum phonation time (MPT) (sec)	*r*	0.026	0.203	0.112
	*P*-value	0.865	0.186	0.469
Mean intensity of sustained phonation (dB)	*r*	−0.165	−0.210	−0.190
	*P*-value	0.295	0.182	0.229
Fraction of locally unvoiced frames (%)	*r*	0.126	0.069	0.105
	*P*-value	0.606	0.779	0.669
Number of voice breaks	*r*	0.062	−0.015	0.028
	*P*-value	0.818	0.956	0.918
Speech rate (syll/s)	*r*	−0.031	0.023	−0.008
	*P*-value	0.842	0.885	0.958
Speech intelligibility (%)	*r*	−0.129	−0.149	−0.142
	*P*-value	0.480	0.417	0.437

## Discussion

The main objective of this study was to determine a relationship between speech and upper extremity bradykinesia in advanced PD patients. We found different correlations between speech acoustic variables and tapping rate in both the defined-OFF and defined-ON medication conditions. However, it is important to keep in mind that the correlation tests, either Pearson or Spearman, do not prove causality but only strength of association. In particular, in our study, neither the methodology employed nor the statistical analysis was designed to infer causation. In addition, curiously, we found a positive correlation between speech impairment and left bradykinesia during the defined-OFF condition and with both left and right bradykinesia during the defined-ON condition, which could be surprising. However, in the defined-OFF condition, the correlation between speech acoustic variables and right tapping showed a trend toward significance. Thus, we may assume that, with a larger cohort, the correlation might become significant even with the right tapping.

In our cohort, the MPT correlated with upper limb bradykinesia in both pharmacological conditions, meaning that patients with a longer MPT performed better on the tapping test. Phonatory alterations are quite common in PD, including insufficient breath support, a reduction in phonation time, increased acoustic noise, instability of the articulatory organs, microperturbations of frequency/amplitude, and a harsh, breathy voice quality (31). The physiological and anatomical correlates of these alterations have been investigated through laryngoscopy, stroboscopy, photoglottography, laryngeal electromyography, computed-tomography, pulmonary function testing, and aerodynamic assessments (32). These correlates have revealed numerous abnormalities, including incomplete glottic closure and vocal fold hypoadduction/bowing to account for these voice changes. Many of these phenomena are likely related to rigidity or bradykinesia of the laryngeal muscles (32).

The clinical feature of hypokinetic dysarthria reflects the effects on the speech of aberrations in the control of proper background tone and the supportive neuromuscular activity on which the quick, discrete, phasic movements of speech are superimposed. Hypokinetic dysarthria prominently affects aspects of speech motor control such as the preparation, maintenance, and switching of motor programs with movements that are attenuated in range and amplitude and restricted in their flexibility and speed, allowing inferences about the role of the basal ganglia control circuit in speech motor control (33).

The MPT, a marker of reduced phonation time, depends on many factors, including phonation volume (which varies with age, sex, and stature), mean airflow rate, comprehension of the task, and maximal effort (34). Reduced MPT has been well documented mainly in PD hypokinetic dysarthria (9, 35–39), probably as a consequence of laryngeal dysfunction or decreased respiratory volume, leading to the development of short phrases and short rushes of speech (6). This hyporespiratory pattern may result from the rigidity and bradykinesia in the respiratory muscles, particularly the intercostal ones (38). In PD patients, reduced respiratory excursions, reduced vital capacity, paradoxical respiratory movements, rapid breathing cycles, and difficulty altering vegetative breathing for speech breathing seem consistent with rigidity, hypokinesia, and difficulty in initiating movements (33). These factors could significantly contribute to reduced physiologic support for speech and some of the phonatory disorders and prosodic abnormalities, including short phrases and short rushes of speech (33).

The short-term improvement of MPT with levodopa was demonstrated previously, supporting the hypothesis that levodopa might improve thoracic mobility in PD patients (38). This finding was also confirmed by a recent study from our group, which showed that MPT was the only speech acoustic variable responsive to levodopa in the acute setting (6). The correlation between phonatory alterations and limb bradykinesia has also been confirmed (based on clinical scales) in *de novo* PD patients (22), suggesting that dopaminergic deficiency may be involved in voice dysfunction in PD, presumably through bradykinesia and/or rigidity of the laryngeal musculature (22).

Based on these premises, we suppose that the correlation found between MPT and upper extremity tapping rate in our cohort might reflect a common pathophysiological basis (i.e., bradykinesia of appendicular, laryngeal, and respiratory muscles) with the involvement of prevalent dopaminergic pathways responsive to levodopa. In fact, the degree of response to levodopa is different between these two parameters, as confirmed in our cohort. Indeed, no correlations were found between levodopa-induced percentage changes in MPT and upper extremity bradykinesia, which means that, even if levodopa improves both parameters, this improvement is not uniform. Moreover, it must be considered that the tapping test and MPT are only two specific findings of two more complex functions such as movement and speech.

We found a weak correlation between speech rate and right tapping in the defined-OFF medication condition. Nonetheless, the absence of correlations in the other defined-OFF and -ON medication conditions would exclude an unequivocal relationship between the two findings.

Speech rate is generally expressed as the number of syllables pronounced during a defined time period. It is affected by different factors such as segment duration, variability between the duration of utterances, and the pause time between the different utterances (40). In PD patients, speech rate alterations have been found in both directions (i.e., slower and faster), and the mean rate differences between PD and control speakers were not found to be significant due to a highly heterogeneous overall group performance (3). Previous studies have also shown little or no effect of levodopa administration or bilateral STN-DBS on speech rate in PD patients (41–43). This was not confirmed in our cohort; indeed, speech rate was significantly reduced after levodopa intake. These contradictory results about the short-term effects of levodopa on speech rate and rhythm indicate a considerable impact of the nondopaminergic mechanism, which are implicated in the impairment of time perception, motor planning, and dysfunctional feedback mechanisms (40).

Our study has several limitations, including the retrospective origin of the data and the lack of a control group of healthy subjects to compare with the PD cohort. In addition, we assessed only advanced PD patients, so further studies are needed to test the relationship between upper limb bradykinesia and speech variables in early PD patients.

Furthermore, we did not collect several demographic and anthropometric variables of the participants, including weight, height, and BMI. In addition, voice features relevant to the acoustic analysis of hypokinetic dysarthria in PD have not been included in the study. It is known that f0, jitter, and shimmer, among other voice features, can be severely impaired in hypokinetic dysarthria, especially in advanced-stage PD. Also, these features are relevant for discriminating early- and advanced-stage PD, so they should always be considered in speech analysis in movement disorders, as nicely reported in Rusz et al. (8) and Suppa et al. (44).

To the best of our knowledge, only one study has compared speech acoustic variables and upper limb motor dysfunction with a quantitative approach, supporting the hypothesis that pathophysiological processes leading to limb motor dysfunction in PD may play a role, at least partially, also in a more complex function such as speech (18). In particular, significant relationships were observed between the quality of voice assessed by jitter and amplitude decrement of finger tapping, consonant articulation evaluated using voice onset time and expert rating of finger tapping, and the number of pauses and Purdue Pegboard Test score (18). Based on their results, Rusz et al. in their study assumed that vocal fold vibration irregularities appeared to be influenced by mechanisms similar to amplitude decrement during repetitive limb movements, while consonant articulation deficits were associated with decreased manual dexterity and movement speed, likely reflecting fine motor control involvement in PD (18). Furthermore, MPT was not included among the different speech variables, and no correlation was found between diadochokinetic rate and markers of upper limb motor dysfunction, as opposed to our cohort (18). This could be explained both by the different tasks used to quantify speech rate (oral diadochokinesis vs. counting from 1 to 20) and the different pharmacological conditions tested in the two cohorts. In fact, our patients were evaluated both in the defined-OFF and defined-ON medication conditions, while in the study by Rusz et al. the patients were evaluated only in chronic pharmacological treatment (18).

## Conclusion

Our study confirms the presence of some correlations between speech acoustic variables and upper extremity bradykinesia in advanced PD patients. This may be due to common pathophysiological mechanisms and the possible involvement of dopaminergic pathways, as assumed for MPT. This confirms the need to take into account independently every single speech parameter altered in PD. As a consequence, speech alterations should not be considered anymore as a solely axial manifestation of the disease unresponsive to dopaminergic treatment and without a relationship with PD cardinal motor symptoms. Future studies will be needed to confirm these data on larger samples and on early-stage PD patients.

## Data availability statement

The datasets for this article are not publicly available due to concerns regarding participant/patient anonymity. Requests to access the datasets should be directed to the corresponding author.

## Ethics statement

The studies involving human participants were reviewed and approved by Comitato Etico dell'Area Vasta Emilia Nord (Protocol Number: 0031287/18), and written informed consent was obtained from participants according to the Declaration of Helsinki.

## Author contributions

FC, GDR, AG, CB, VF, SC, EMe, FA, and FV were responsible for writing the manuscript, data collection, and analysis. FC, GDR, CB, AG, SC, VF, EMe, SP, EMo, FA, and FV were responsible for manuscript drafting. CB, SP, EMe, FA, and FV were responsible for manuscript revision. All authors have read and approved the final manuscript.
